# Does the Electronic Health Card for Asylum Seekers Lead to an Excessive Use of the Health System? Results of a Survey in Two Municipalities of the German Ruhr Area

**DOI:** 10.3390/ijerph16071178

**Published:** 2019-04-02

**Authors:** Pia Jäger, Kevin Claassen, Notburga Ott, Angela Brand

**Affiliations:** 1 Section for Social Policy and Social Economy, Faculty of Social Sciences, Ruhr-University Bochum, Universitätsstr. 150, 44801 Bochum, Germany; notburga.ott@rub.de; 2 Department of International Health, Faculty of Health, Medicine and Life Sciences, Maastricht University, Duboidomein 30, 6229 CT Maastricht, The Netherlands; a.brand@maastrichtuniversity.nl; 3 Department of Medicine, Faculty of Health, Witten/Herdecke University, Alfred-Herrhausen-Straße 50, 58448 Witten, Germany; kevin.claassen@uni-wh.de

**Keywords:** refugee health, electronic health insurance card, prevention and medical care of refugees, access to healthcare, refugees’ use of outpatient and inpatient medical care

## Abstract

Background: The initial and intermediate-term access of refugees to healthcare in Germany is limited. A previous study showed that the obligation to request healthcare vouchers at the social security offices decreases the asylum seekers’ consultation rate of ambulant physicians. The introduction of the Electronic Health Insurance Card (EHIC) for asylum seekers is considered skeptically by some municipalities and federal states, among other reasons due to the fear of an overuse of health care services by asylum seekers. The aim of this study is to further evaluate the data of the authors’ initial study with a new focus on inpatient care as well as a differentiation of the ambulant consultation rate into general practitioners and outpatient specialists. Methods: The now-differentiated consultation rate of the initial study as well as the asylum seekers’ use of inpatient care are compared to the values of the sex- and age-corrected autochthonous population as given by the German Health Interview and Examination Survey for Adults (DEGS1). A mean difference test (student’s t-test) is used for comparison and significance testing. Results: Asylum seekers who were in possession of the EHIC were significantly less likely to visit their ambulant general practitioners and specialists than the German autochthonous population. Simultaneously, this difference is partly compensated for by their more frequent use of impatient care. Conclusions: There is no indication that the EHIC leads to an overuse of healthcare services.

## 1. Introduction

Especially as a result of the armed conflicts in the Middle East, Germany has experienced an increased number of applications for asylum since 2014. The German Federal Office for Migration and Refugees received 476,649 applications for asylum in 2015. In 2016, the number of applications for asylum reached a peak with 745,545 applications. It should be noted that a few months may elapse between the arrival and the application for asylum, so that the number of applications does not necessarily correspond to the number of people arriving. Since 2017, the number of applications for asylum has been decreasing again: 222,683 people applied for asylum in 2017 and 110,324 between January and July 2018. [1]

The distribution of refugees within Germany takes place in accordance with the so-called “Königsstein Key”. This distribution quota is calculated annually by the Federation-Länder Commission on the basis of the population number and the tax income with the aim of a suitable and fair distribution. With 21.14%, the federal state North Rhine-Westphalia had the highest distribution quote [2].

The arrival of refugees on this quantitative level brought major challenges for the federal states and municipalities [3]. The situation is called publicly a “refugee crisis” [4]. In 2017, nationwide 1 million inhabitants met 2.4 first-time asylum seekers in Germany; as a comparison: it is 1.2 in the whole European Union and 5.3 in Greece but 164,000 in Lebanon and 43,000 in Turkey [5,6].

Although the actual proportion of refugees within the total population is not that high by international comparison, this migration and escape movement after 2014 had a massive impact on the German society and politics [7].

Particularly challenging and controversial is the health care of asylum seekers. For asylum seekers in Germany, only acute health care is granted (§ 4 Abs. 1 AsylbLG). After staying for 15 months, they receive services that are equal to those of the autochthonous population (§ 2 Abs. 1 AsylbLG). Before that, they have to ask for health care vouchers at the particular social security office in many of the German federal states. In North-Rhine Westphalia (NRW), the situation since 2016 is inasmuch unique as the municipalities are free to introduce the Electronic Health Insurance Card (EHIC) for asylum seekers before the end of the 15 months (§ 264 Abs. 1 SGB V). Recently, 23 of 396 NRW municipalities exercised this right. Although the services remain de jure on the level of acute health care, it seems that the introduction of the EHIC for asylum seekers increases their consultation rate of ambulant physicians [8].

The introduction of the Electronic Health Insurance Card (EHIC) is discussed controversially in Germany. Although the database is thin overall, because representative, valid data is lacking [9], initial research indicates that the EHIC is improving the medical care of asylum seekers [8]. Many doctors demand a nationwide introduction of the EHIC for all refugees [10].

Administration costs and an increase of medical care costs are feared by critics [11]. Previous research indicates that the costs of excluding asylum seekers and refugees from health care appears to be ultimately higher than granting regular access [12]. Critics also fear that the EHIC could represent another migration motive for refugees to come to Germany or lead to an abuse or an excessive use of the German health care system [13]. While initial studies on the economic aspects of the card are available, a valid comparison between the refugees’ use of the German health care system and the German reference population is still missing.

The aim of this study is to further evaluate the data of the authors’ initial study on the use of the health care system by asylum seekers who possess an EHIC in Germany [8] and to compare this use to the sex- and age-correlated autochthonous population with a new focus on inpatient care as well as a differentiation of the consultation rate into general practitioners and outpatient specialists.

## 2. Materials and Methods

One hundred and forty-six (146) interpreter-supported standardized interviews in German, English, French, Arabic, and Farsi with asylum seekers living in two municipalities in North-Rhine-Westphalia that have introduced the EHIC were analyzed. All interviews took place in between the years 2016 and 2017 in cooperation with the municipal administration and the municipal health department in the domestic environment of the respondents. By the time of the survey, 88.36% of the respondents lived in a community camp and 11.64% in an own department.

The survey was communicated ex ante by the officials, the respondents were partly approached by the interpreters, and participation was voluntary. Every respondent provided informed consent by signature. Ethical approval for this study was provided by the Bochum ethical review committee (reference number 16-5920).

Data were obtained on the duration of the stay in Germany until the survey was taken as well as the duration of the period in which the asylum seekers were in possession of the EHIC. The respondents were asked for their number of visits to the doctor, whereby a distinction was made between general practitioners and specialists. Visits to volunteering doctors were also taken into account. From this, the consultation rate (CR) of ambulant physicians during the period in which the respondents were in possession of the EHIC was determined as the main variable of interest. If a CR could not be identified due to lack of information, the corresponding cases were not considered further, so that 75 cases in total could be analyzed.

Age, sex, and other important personal information, such as the country of origin, marital status, number of children, and educational attainments, were surveyed. Additionally, medication, a self-rating of German language skills, and the presence of chronic diseases—whereby the most common ones [14] were covered—were asked for.

A deeper explanation of the data generation regarding the asylum seekers is to be found in the study design section of the previous paper [8]. Note that the 146 respondents who are the basis of the study on hand are a subset of the existing dataset without those asylum seekers who still had to use healthcare vouchers from the social security office. Covariables, which are only used to describe the sample, were collected and in part already analyzed previously and in other respects.

The determined CR of general practitioners and specialists by refugees was compared to the one of the total population in Germany. The basis are the results of the German Health Interview and Examination Survey for Adults (DEGS1) of the Robert-Koch-Institut (RKI) on the utilization of outpatient and inpatient health services in Germany (*n* = 7988) [15]. The comparison is sex- and age-adjusted for the age-groups 18–29 years, 30–39 years, 40–49 years, 50–59 years, 60–69 years, and 70–79 years.

A mean difference test (student’s t-test) was used to compare the total male, and female group of interviewed asylum seekers to the German reference population. For the sex-adjusted age groups with a sufficient number of cases (with a minimum of 20), a t-test in order to compare them to the sex- and age-adjusted German reference group was also conducted. The age-and sex-adjusted groups of this survey that do not have a sufficient number of cases for a statistical analysis are presented descriptively. In addition, a graphical representation is given.

## 3. Results

The following section is subdivided. The results for the asylum seekers of the authors’ study (3.1), the German age- and sex-adjusted reference population given by the DEGS1 sample of the RKI (3.2), and the comparison between the two groups (3.3) are presented.

### 3.1. Asylum Seekers

With 80.2%, the 146 surveyed asylum seekers were predominantly male. The average age was 30.86 (95% CI 29.19–32.48) years; 29.96 (95% CI 28.38–31.54) years for men and 35.05 (95% CI 29.16–40.93) years for women.

The observation period in which the respondents were in possession of the EHIC was 1471.5 months in total and 11.68 (95% CI 10.27–13.09) months per capita. The respondents had already stayed 18.71 months in Germany on average (95% CI 16.96–20.50) with a minimum of less than one month and a maximum of 51 months. They had been for 8.86 months (95% CI 7.68–10.07) in Germany without the EHIC, either because they were waiting for it or because they were in another commune that did not use the EHIC for the medical care of refugees.

Of the respondents, 57.53% were single, 37.67% were married, 2.74% were divorced, and 2.05% were widowed. With 63.45%, the majority was without children, 11.03% had one child, 9.66% two children, and 15.86% three or more children. According to self-reported data, 37.67% of the interviewed refugees were without education or vocational qualification, 3.42% had completed vocational training, 28.77% had a general university entrance qualification, and 15.75% a university degree. In the self-assessment, 4.79% had “very good”, 23.29% “good”, 34.93% “mediocre”, 21.23% “bad”, and 15.75% “very bad” German language skills.

With regard to pre-existing diseases, 37.24% reported to suffer from at least one chronic disease. In detail, diabetes was mentioned by six persons, cardiovascular diseases by one person, psychiatric or psychosomatic disorders by 21 persons, back problems by 14 persons, joint diseases by seven persons, cancer diseases by two persons, thyroid diseases by five persons, and other unspecified diseases, such as complaints after a surgical operation or unspecific pain, by 29 persons. The psychiatric or psychosomatic disorders were most frequently reported by at least 14.38% of the respondents followed by unspecific diseases, whereby a high correlation between the two can be assumed with unspecific diseases representing a comorbidity. In their country of origin, 17.52% of the interviewed asylum seekers had been on medication. After their arrival in Germany, a medication treatment was obtained by 29.41% of the respondents. A total of 14 people received medication in Germany who had not been on medication in their country of origin; four people had been on medication in their country of origin without continuing the treatment in Germany.

A total number of 75 persons could answer the question of visits to the doctor. For 72 of these cases, information on age was available.

A general practitioner was visited by 57.33% of the respondents since their arrival in Germany. The CR is 0.23 (95% CI 0.14–0.33) per month, which means 2.81 (95% CI 1.71–3.92) per year on average. Other ambulant doctors, such as volunteering doctors in the camps, were visited by 10.81% of the interviewed asylum seekers overall with a CR of 0.02 (95% CI 0.00–0.03) visits per month on average and 0.19 per year (95% CI 0.00–0.38). Adding visits to general practitioners and other ambulant doctors together, the CR is 2.94 visits per year (95% CI 1.81–4.07), which corresponds to 0.25 per month (95% CI 0.15–0.34).

An ambulant specialist was visited by 72% of the asylum seekers. The average of each person is 2.29 visits per year (95% CI 1.55–3.04), which corresponds to 0.19 per month (95% CI 0.13–0.25).

An inpatient treatment was used by 36% of the respondents since their arrival. Within the period of one year, 27.05% used inpatient medical services, such as in a hospital or hospital ambulance (in men 31.4% and in women 8.37%). Important information on the respondents (*n* = 146, for *n* = 128 of them information on age is available) differentiated by age groups is presented in Table 1 (NA = not available).

### 3.2. Reference Population

In the following section, the situation of the autochthonous German population is presented.

Overall, 12.9% of the German inhabitants used an inpatient medical service within the last 12 months according to the DEGS1 results of the RKI that are used as a reference.

In the group of 18–29 years old people, the CR of general practitioners in the last 12 months is 7.6 on average (95% CI 7.0–8.2). That of persons aged 30–39 is 7.7 (95% CI 7.1–8.4), aged 40–49 8.4 (95% CI 7.8–9.0), aged 50–59 9.9 (95% CI 9.2–10.6), aged 60–69 11.9 (95% CI 10.8–12.9), and aged 70–79 11.5 (95% CI 10.8–12.5). All in all, the CR is 9.2 (95% CI 8.9–9.5).

The CR of the reference group for specialists is on average 3.0 (95% CI 2.9–3.1) in the group of respondents aged 18–29 years. It is 3.0 (95% CI 2.9–3.2) within the group aged 30–39, 3.3 (95% CI 3.2–3.5) in the group aged 40-49, 3.7 (95% CI 3.5–3.8) in the group aged 50–59, 4.2 (95% CI 4.0–4.3) in the group aged 60–69, and 4.3 (95% CI 4.1–4.4) in the group aged 70–79.

The CR of general practitioners is higher in women than in men and increasing with age for general practitioners, specialists, and inpatient medical care. This must be considered within the comparisons, since the survey population is younger on average than the reference population of DEGS1 and also has no homogenous sex distribution in contrast to the reference population.

### 3.3. Comparison

**Figure 1:**
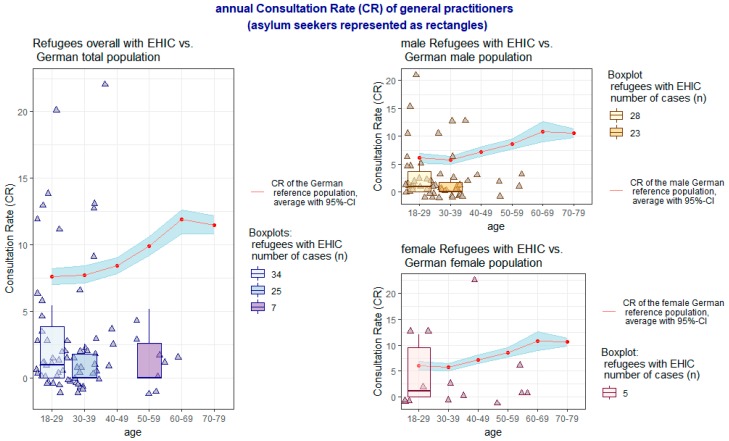
Annual Consultation Rate (CR) of general practitioners.

**Figure 2:**
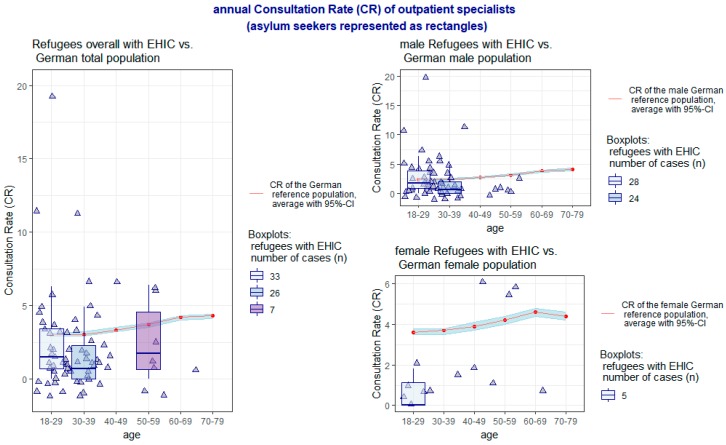
Annual Consultation Rate (CR) of outpatient specialists.

Taking these aspects into account, the individual results of the comparison of the CR for general practitioners and outpatient specialists between the interviewed sample and the DEGS1 reference group (the average is represented as a red line with surrounding CI) are presented age-correlated for each group of men and women in Figure 1 and Figure 2. The CR of the survey sample is shown as triangles; in the age groups with at least five persons a supplementary boxplot is added.

Overall, the CR was significantly lower in the interviewed sample of asylum seekers for general practitioners (*p* < 0.001, 95% CI 1.71–3.92), even with the inclusion of visits to “other ambulant doctors” (*p* < 0.001, 95% CI 1.81–4.07), as well as for specialists (*p* = 0.0019, 95% CI 1.55–3.04). With regard to sex aspects, these significant differences exist both in the CR of general practitioners, including “other ambulant doctors”, by both sexes (*p* < 0.001, 95% CI 1.48–3.71 in men and *p* = 0.0039, 95% CI 0.49–8.28 in women) as well as in the CR of specialists by women (*p* = 0.0155, 95% CI 0.66–3.57). The CR of specialists by men does not differ significantly between the respondents and the DEGS1 reference group.

In contrast, the rate of the respondents using at least one time in 12 months an inpatient medical service is significantly higher than in the reference group of the DEGS1 study (*p* = 0.0047, 95% CI 0.17–0.37). This difference is only existent in men (*p* = 0.00195, 95% CI 0.2–0.43) and not in women (*p* = 0.4, 95% CI −0.04–0.2).

## 4. Discussion

In the previous investigation, an increased utilization of outpatient medical services by asylum seekers who are in possession of the EHIC compared to those receiving healthcare vouchers was shown, while the possession of the EHIC appeared to be an independent influencing factor on the use of medical care. From this, it is concluded that the supply via healthcare vouchers at the social security office could be a relevant barrier for asylum seekers [8]. In addition to administrative and utility costs, fears in politics and within the population are a possible cause of a supply via healthcare vouchers, whereby above all an overuse of medical services is feared.

In this survey, the CR of general practitioners and specialists by asylum seekers who are in possession of an EHIC are significantly below the CR of the DEGS-1 survey sample of the RKI used as a reference group. Comparing these groups with regards to age and sex, this effect can be demonstrated for almost all groups. In this survey, the feared overuse of medical services cannot be shown. Much more likely, the use of medical services by asylum seekers with the EHIC seems to approach a needs-adapted care.

Other studies have reported increased use of inpatient services by migrants in Germany [16]. At the same time, a lower level of health care utilization is seen as a result of both an unequal access to the health care system and an unequal use [17]. An increased use of inpatient services could be caused by a different behavior, e.g., due to a diverging health care system in the country of origin or barriers in outpatient care; but also an even higher rate of emergencies resulting from a lack of preventive or early outpatient care has to be taken into consideration.

Previous surveys of the refugee population also showed a reduced utilization of medical care services, especially in the outpatient setting, while inpatient treatment increased simultaneously [18]. The study on hand presents further evidence that refugees with the EHIC, like other migrants, indeed receive inpatient treatment more often than the autochthonous German population. In this context, the results of this survey appear plausible.

### Scientific Limitations

The limiting factor of this survey is the low number of cases as well as a high rate of missing values concerning the information on the use of medical services. Additionally, in contrast to the DEGS1 reference group, the survey group had no homogenous age and sex distribution. The number of cases is besides the regional scope one of the main reasons this study does not claim to be representative, but would like to give first indications of the influence of the introduction of the EHIC for asylum seekers in Germany. Further longitudinal studies with a representative sample enabling an accurate analysis based on a representative age and sex adjustment are desirable. This could also allow for an adjustment in terms of further socio-demographic aspects and a better analysis of the inpatient treatment. This was only possible to a limited extent due to the fact that the RKI questioned the one-year prevalence of inpatient treatment and the hospital stay while this survey asked about the use of inpatient treatment since the arrival in Germany with respect to the reception of the EHIC. As a further possible bias, the fact that the reference group may also contain refugees as well as an increased use of medical services following the reception of the EHIC after a long waiting period have to be considered.

Also, distinguishing visits to general practitioners and internists (specialists in internal medicine) who can both work as specialists as well as in primary care (function of a family doctor/general practitioner) is difficult, both in the survey and the reference group. The two selected municipalities for sampling are located in the Ruhr area. While due to personal contacts the otherwise difficult entry was eased, it needs to be stated that these municipalities are possibly not representative of Germany as a whole, especially if we consider that there are regions with a much smaller density of medical institutions. The study is, despite its quantitative methods, rather explorative than affirmative within the broader topical field of refugee health.

## 5. Conclusions

Although the CR of general practitioners and specialists is significantly higher in asylum seekers with the EHIC than in those receiving health care vouchers, which was found in the previous analysis, even asylum seekers in possession of the EHIC were significantly less likely to seek the outpatient medical care of general practitioners and specialists than the German general population. There are no indications of an overuse of medical services among asylum seekers, while, on the other hand, there is much evidence for improvements in the medical care and a reduction of barriers due to the possession of the EHIC. The aforementioned trend of a more frequent use of inpatient treatment by migrants seems to persist in asylum seekers.

## Figures and Tables

**Table 1:** Important information on the respondents differentiated by age groups.

Age Groups	18–29	30–39	40–49	50–59	60–69	70–79
**Absolute number**	60	45	13	9	1	0
**Hereof men absolute, (%)**	52, (86.67%)	39, (86.67%)	9, (69.23%)	6, (66.67%)	0	0
**Presence of chronic diseases (any)**	19, (32.20%), (1 NA)	14, (31.11%)	8, (61.54%)	6, (66.67%)	1, (100%)	NA
**Mean CR/year of general practitioners (95% CI), (number of NA)**	3.18, (1.44–4.91), *n* = 34, (26 NA)	2.06, (0.48–3.65), *n* = 26, (19 NA)	7.29, (−9.39–23.97), *n* = 4, (9 NA), (1 outlier)	1.48, (−0.40–3.35), *n* = 7 (2 NA)	1.8, *n* = 1, (0 NA)	NA
**Mean CR/year of outpatient specialists (95% CI), (number of NA)**	2.63, (1.24–4.01), *n* = 33, (27 NA)	1.78, (0.69–2.86), *n* = 26 (19 NA)	2.66, (−1.0–6.31), *n* = 4, (9 NA), (1 outlier)	2.65, (0.23–5.10), *n* = 7, (2 NA)	0, *n* = 1, (0 NA)	NA
**Use of inpatient services at least one/year in %, *n*, (NA)**	26.92, *n* = 34, (26 NA)	26.81, *n* = 26, (19 NA)	24.63, *n* = 4, (9 NA)	36.73, *n* = 7, (2 NA)	60.0, *n* = 1, (0 NA)	NA

## Abbreviations

EHIC	Electronic Health Insurance Card
CR	Consultation Rate of Ambulant Physicians
RKI	Robert-Koch-Institute
DEGS-1	German Health Interview and Examination Survey for Adults
NA	Not available
CI	Confidence Interval
